# Interpretable AI for bio-medical applications

**DOI:** 10.20517/ces.2022.41

**Published:** 2022-12-28

**Authors:** Anoop Sathyan, Abraham Itzhak Weinberg, Kelly Cohen

**Affiliations:** 1 Department of Aerospace Engineering, University of Cincinnati, Cincinnati, OH 45231, USA.; 2 Department of Management, Bar-Ilan University, Ramat Gan 5290002, Israel.

**Keywords:** Explainable AI, LIME, SHAP, neural networks

## Abstract

This paper presents the use of two popular explainability tools called Local Interpretable Model-Agnostic Explanations (LIME) and Shapley Additive exPlanations (SHAP) to explain the predictions made by a trained deep neural network. The deep neural network used in this work is trained on the UCI Breast Cancer Wisconsin dataset. The neural network is used to classify the masses found in patients as benign or malignant based on 30 features that describe the mass. LIME and SHAP are then used to explain the individual predictions made by the trained neural network model. The explanations provide further insights into the relationship between the input features and the predictions. SHAP methodology additionally provides a more holistic view of the effect of the inputs on the output predictions. The results also present the commonalities between the insights gained using LIME and SHAP. Although this paper focuses on the use of deep neural networks trained on UCI Breast Cancer Wisconsin dataset, the methodology can be applied to other neural networks and architectures trained on other applications. The deep neural network trained in this work provides a high level of accuracy. Analyzing the model using LIME and SHAP adds the much desired benefit of providing explanations for the recommendations made by the trained model.

## 1. INTRODUCTION

In recent years, we have witnessed growth in the usage and implementation of machine learning based decision making and predictive analytics. Practically speaking, machine learning models are ubiquitous^[1]^. One of the reasons for this growth is the contribution of machine learning to their users and decision makers. In recent times, there has been a rise in the development of new computational infrastructures such as cloud storage and parallel computation^[2]^, which has contributed to faster training of the models. Many papers contribute to the effort of developing machine learning models that excel in metrics such as accuracy, efficiency and running time. The more complex models are usually more accurate^[3,4]^. However, the ability of humans to understand it is negatively correlated to model complexity^[5]^. One of the challenges to eXplainable AI (XAI) is its implementation in real-life applications. XAI has inherent challenges such as lack of expertise, inherently biased choices, lack of resiliency for data changes, algorithms and problems interference challenges, local context dependency of the explanations and lack of causality of explanations between input and output^[6]^. These challenges intensify for clinical and medical real-life use cases such as in the breast cancer use case we consider in this work. In order to overcome these challenges, there is a need for a strong interaction between the XAI system and the decision makers. In our case, the domain experts, radiologists and physicians need to examine the XAI results and add their own perspectives based on their prior knowledge before making final decisions. In addition, they can add their feedback in order to improve and fine-tune the XAI system. Another way to increase the trustworthiness of the XAI can be synergy between different XAI approaches and algorithms. In our case, we use Local Interpretable Model-Agnostic Explanations (LIME) and Shapley Additive exPlanations (SHAP). Each of them has a different approach to extract the explanations of the model predictions. When both XAI approaches provide the same or similar results, it is an indication that the user can have higher confidence in the interpretability of the model.

To realize the immense economic and functional potential in AI applications that have stringent safety and mission critical requirements in areas such as healthcare, transportation, aerospace, cybersecurity, and manufacturing, existing vulnerabilities need to be clearly identified and addressed. The end user of such applications as well as the taxpaying public will need assurances that the fielded systems can be trusted to deliver as asked. Moreover, recent developments evaluating the trustworthiness of high-performing “black-box” AI have classified them using the term “Brittle AI”, as a retrospective look at DARPA’s explainable AI program. These developments coupled with a growing belief in the need for “Explainable AI” have led major policy makers in the US and Europe to underscore the importance of ”Responsible AI”.

Recently, on June 28, 2022, a group of Cruise robotaxis abruptly stopped working on a street in San Francisco, California, which caused traffic to stop for several hours until employees of the company arrived. Cruise, which is backed by General Motors and Honda, has been testing its technology in San Francisco since February, but only launched a commercial robotaxi service a week prior to this malfunction. The cars have no human driver at all but operate under certain restrictions (good weather and a speed limit of 30mph). They only offer the taxi service in a dedicated area of the city during after-hours between 10PM and 6AM^[7]^. While no one was hurt in this instance, several questions are raised concerning the maturity of the autonomous system technology and the need to ensure that these autonomous systems operate as intended. The outcome is that the public is concerned and does not trust such systems. In order to handle such events in future, we can find several approaches in literature. Some of the methods include observer fault estimation based on sensors^[8]^, nature optimal control systems^[9]^ and predictive control models^[10]^. All the approaches add a layer to the system that is supposed to detect any faulty behavior of the system. The mission in such cases is to translate the predictions of the control systems into a way that its operators and decision makers will be able to understand. The system has to provide a way to explain what happened and what action has to be taken by humans. This is one of the deliverables that XAI is supposed to yield.

According to the National Institute of Standards and Technology (NIST)^[11]^, determining that an AI system is trustworthy just because all system requirements have been addressed is not enough to guarantee widespread adoption of AI. Moreover, according to NIST, “It is the user, the human affected by the AI, who ultimately places their trust in the system,” and furthermore, “alongside research toward building trustworthy systems, understanding user trust in AI will be necessary to minimize the risks of this new technology and realize its benefits.

In June 2022, Kathleen Hicks, Deputy Secretary of Defense, released a report that clarifies the DoD perspective concerning trust in AI systems as follows: “The Department’s desired end state for Responsible AI (RAI) is trust. Trust in DoD AI will allow the Department to modernize its warfighting capability across a range of combat and non-combat applications, considering the needs of those internal and external to the DoD. Without trust, warfighters and leaders will not employ Al effectively and the American people will not support the continued use and adoption of such technology”^[12]^. This paradigm shift in policy will have a major impact on the continued development and fielding of AI systems for DoD and for the safety critical systems in the civilian arenas such as health, energy transportation etc.

In line with DoD’s perspectives on trust in AI, it is important that users of AI models be able to assess the model, its decisions and predictions by their ability to understand it. In addition, for better understanding, the users would like to get answers to questions such as what needs to be done to change the model or its prediction. This is one of the motivations for the rapid growth in popularity of the paradigm called XAI. The interaction between machine learning models and their users has become one of the crucial points in usage and implementation of AI systems. Many emerging algorithms try to solve this human-machine interaction by providing a meaningful explanation for the model.

There are ways to classify the XAI approaches by several criteria^[13]^ such as: model dependency, sample particularity, explainability timing and the interaction between the explanation to the model itself. More specifically, independence of the explainability of the model itself is called model agnostics. The explanation of the entire model is called global explainability, while explaining a particular sample is called local explainability. The position of the explainability process in model life cycle determines whether the explainability is pre-model, in-model or post-model.

This paper uses two popular approaches for XAI: LIME^[14,15]^ and SHAP^[16]^. Both are attribution-based explanation models. Attribution-based explanation models find and quantify the most contributed features on model predictions. In addition, both models are relatively easy to use, and their results can be plotted and easily interpreted. LIME and SHAP in our case are used as Post-hoc models, locally interpretable and model agnostic. Although both LIME and SHAP explain the predictions made by the trained model, they use different approaches. SHAP relies on Shapley values for finding the best contributing features^[16]^, while LIME explains the model decision in a local region around a particular sample^[14]^. Each approach has its own benefits. Using both approaches supports the explainability level of our deep learning model. Using both LIME and SHAP allows us to compare the insights gained using the two tools. Additionally, since the two tools work independently of each other, the commonalities between the insights gained can be used to gain a better understanding of the trained model as well as how the different features play a role in the diagnosis/prediction.

## 2. XAI FOR HEALTHCARE

The implementation of XAI for increasing trustworthiness can also be found in biomedical studies such as drug-drug interactions prediction^[17]^ as well as classification of protein complexes from sequence information^[18]^. In our case, we use the XAI for the interpretability of breast cancer predictions. The combination of the two has a fast-growing demand^[2]^. The benefits of implementing XAI in medical fields provide opportunity for prevention and better treatment^[2]^. The XAI helps clinicians in the diagnostic process as well as their recommendations^[2]^. This in turn helps the patients to trust the model results and system recommendations. This can also increase the probability that the patient will accept and follow the recommended medical treatment. Moreover, XAI can decrease the probability of error in the diagnostic process since it helps clinicians to focus on the relevant data and help them to better understand the model recommendations.

XAI is an evolving field. As mentioned before, at this current stage, even state-of-the-art XAI algorithms have disadvantages. In literature, we can find approaches that aim to improve some aspects. One of the main challenges of using XAI in healthcare environments is the need to remain neutral regarding preferences. We can find a bona fide approach called scientific explanation in AI (sXAI) that can be used in the field of medicine and healthcare^[19]^. An additional approach based on integrated Electronic Medical Records (EMR) medical systems is described in^[20]^. The approach focuses on explainability and interoperability from the human aspect. Ensemble of machine learning (ML) can also increase the level of interpretability, as can be seen in^[21]^. In^[21]^, the author use ensemble of ML for logic driving of anthropometric measurements influencing body mass index (BMI). Additional evidence for the implementations of several XAI models is mentioned in^[22]^. The paper shows how integrating XAI models helps to increase the persuasive and coherence levels in the decision making of clinicians and medical professionals teams. The usage of XAI has shown an improvement in transparency and reliability in the field of neuroscience field^[23]^.

In this paper, we apply some XAI concepts to a use case applicable to the medical field. Our work focus on XAI implementation for breast cancer diagnostics. Our research uses the commonly researched UCI breast cancer dataset. We focus on breast cancer since it is the most common type of cancer amongst women^[24]^. The usage of XAI for diagnostics and prediction of breast cancer can impact and help a large number of patients. The UCI breast cancer dataset includes 569 data points^[25]^. Each data point consists of 32 attributes that include the ID number, the diagnosis, and 30 features used as predictors in this work. The 30 predictors include the mean, standard deviation and the mean of 3 largest values of 10 features: (1) radius (mean of distances from center to points on the perimeter); (2) texture (standard deviation of gray-scale values); (3) perimeter; (4) area; (5) smoothness; (6) compactness; (7) concavity; (8) concave points; (9) symmetry; and (10) fractal dimension.

## 3. METHODOLOGY

### 3.1. LIME

LIME is one of the methodologies that is used to explain the predictions made by machine learning classifier models^[26]^. It can explain individual predictions made by text classifiers as well as classifiers that are modeled on tabular data.

In this work, we are focusing on using LIME to explain decisions made by a neural network classifier that works on tabular dataset. The process of LIME to explain individual predictions are as follows:

1. For each instance that needs to be explained, LIME perturbs the observation *n* times.
2. For tabular data, the statistics for each variable in the data are evaluated.
3. The permutations are then sampled from the variable distributions within the neighborhood of the original data point for which an explanation is being sought.
4. In our case, the original model is a neural network. The trained neural network model is used to predict the outcome of all permuted observations.
5. Calculate the distance from the perturbed points to the original observation and then convert it to a similarity score.
6. Select *m* features best describing the original model outcome for the perturbed data.
7. Fit a simple model (linear model) on the perturbed data, explaining the original model outcome with the *m* features from the permuted data weighted by its similarity to the original observation.
8. Extract the feature weights from the simple model and use these as explanations.

### 3.2. SHAP

SHAP is another methodology used for obtaining explanations for individual predictions. Additionally, SHAP can provide additional insights into predictions made across a set of data points. SHAP is based on Shapely values, a concept that is derived from game theory^[16]^. This is a game theoretic approach to explain any predictions made by a machine learning model. Game theory deals with how different players affect the overall outcome of a game. For the explainability of a machine learning model, SHAP considers the outcome from the trained model as the game and the input features that are used by the model as the players. Shapley values are a way of representing the contribution of each player (feature) to the game (prediction).

Shapley values are based on the concept that each possible combination of features has an effect on the overall prediction made by the model. The SHAP process for explaining predictions is as follows^[27]^:

1. For a set of *p* features, there are 2^*p*^ possible combination of features. For example, a dataset that consists of three input features (*x*_1_, *x*_2_, *x*_3_) will have the eight possible combinations: (a) no features, (b) *x*_1_ (c) *x*_2_, (d) *x*_3_, (e) (*x*_1_, *x*_2_), (f) (*x*_2_, *x*_3_), (g) (*x*_1_, *x*_3_), (h) (*x*_1_, *x*_2_, *x*_3_).
2. Models are trained for each of the 2^*p*^ combinations. Note that the model that uses no features just outputs the mean of all output values in the training data. This is considered as the baseline prediction (*y*_*ϕ*_).
3. For the data point whose output needs to be explained, the remaining 2^*p*^ − 1 models are evaluated.
4. Marginal contribution of each of the models. Marginal contribution of model-j is calculated using the difference between the predictions made by model-j and the baseline prediction.
(1) $$MC_{j}={\tilde{y}}_{j}-y_{\phi }$$
5. To obtain the overall effect of a feature on the prediction, the weighted mean of the marginal contributions of every model containing that feature is evaluated. This is called the Shapley value of the feature for the particular data point.

### 3.3. Deep neural network

**Figure 1.**
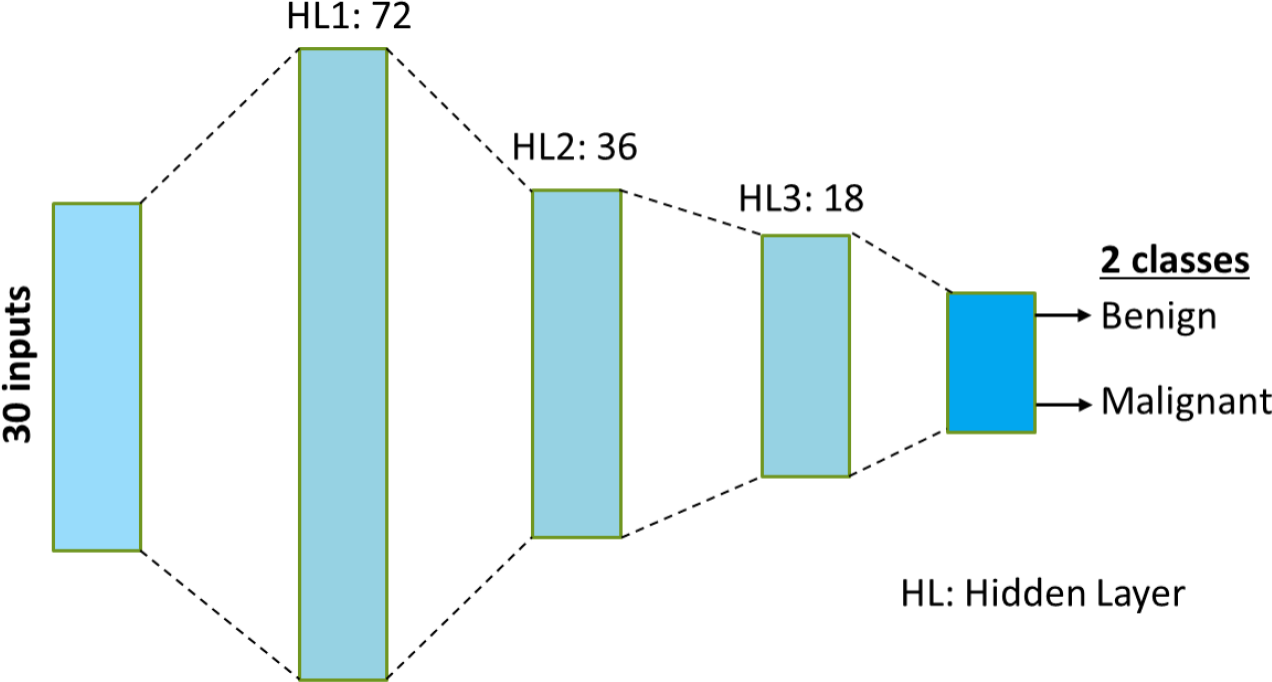
Schematic of the DNN used for classification into benign and malignant. The network uses 30 features and has three hidden layers (HL).

We use a deep neural network (DNN) to diagnose a patient into two classes: benign or malignant. The architecture of the DNN is shown in Figure 1. It uses the 30 features mentioned before to make predictions. The development and training of the DNN was done in PyTorch^[28]^. Rectified linear units (ReLU) are used as the activation functions in the hidden layers, and softmax activation is used at the output layer to output the probabilities to the two output classes.

## 4. RESULTS & DISCUSSION

**Figure 2.**
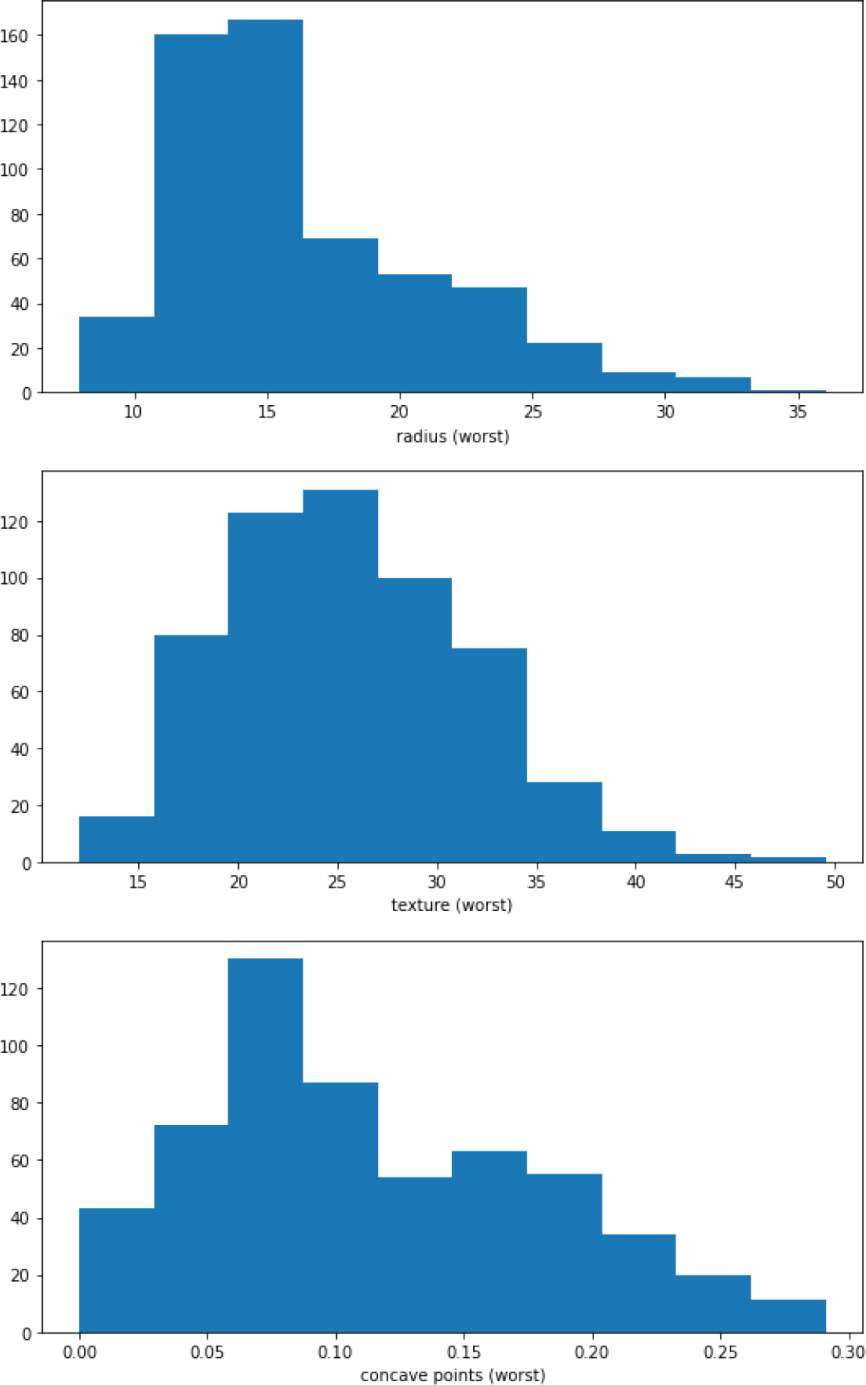
Histograms for three of the important features: radius (worst), texture (worst) and concave points (worst)

The UCI Breast Cancer Wisconsin dataset used an 80%–20% split. This means 80% of the data were randomly chosen for training and the remaining 20% was used for testing. To highlight the data distribution, histograms are shown for three of the important input features in Figure 2.

Since this is a classification problem, cross entropy was used as the loss function. Adam optimizer was used with a learning rate of 0.001 for training the DNN. A batch size of 32 was used when modifying the parameters during the optimization. The DNN was trained on 100 epochs and the trained DNN provided an accuracy of 97% on the test data. This is on the higher end of performance among models trained on this dataset, with the best accuracy noted for this dataset to be 98.6%^[29]^. It is to be noted that this work is not focused on the performance of DNN in terms of accuracy, but instead on explaining the decisions or predictions made by the trained DNN. The trained DNN is further analyzed using LIME and SHAP to understand and explain its predictions.

### 4.1. Results with LIME

**Figure 3.**
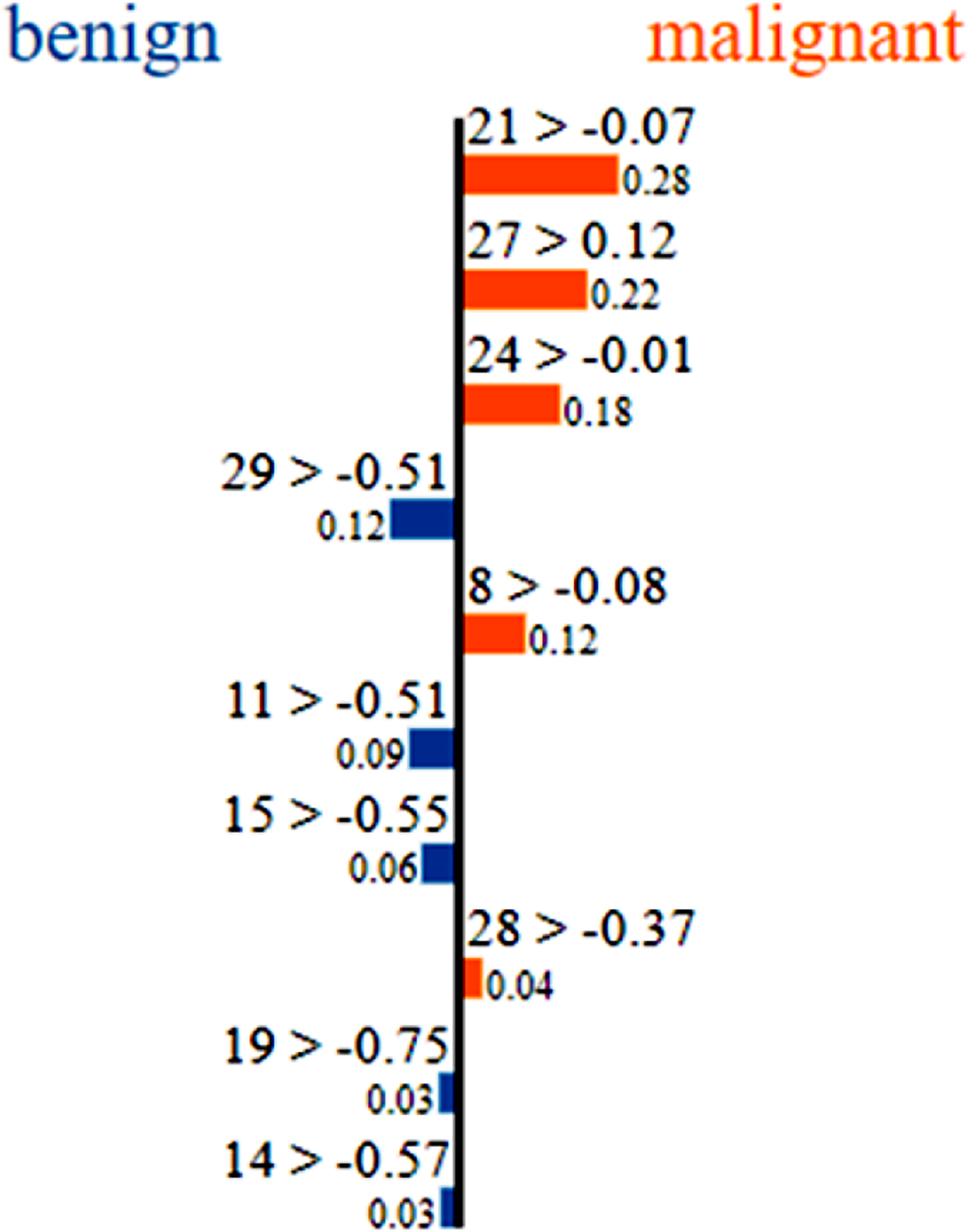
LIME output for a data point that is classified as malignant.

**Figure 4.**
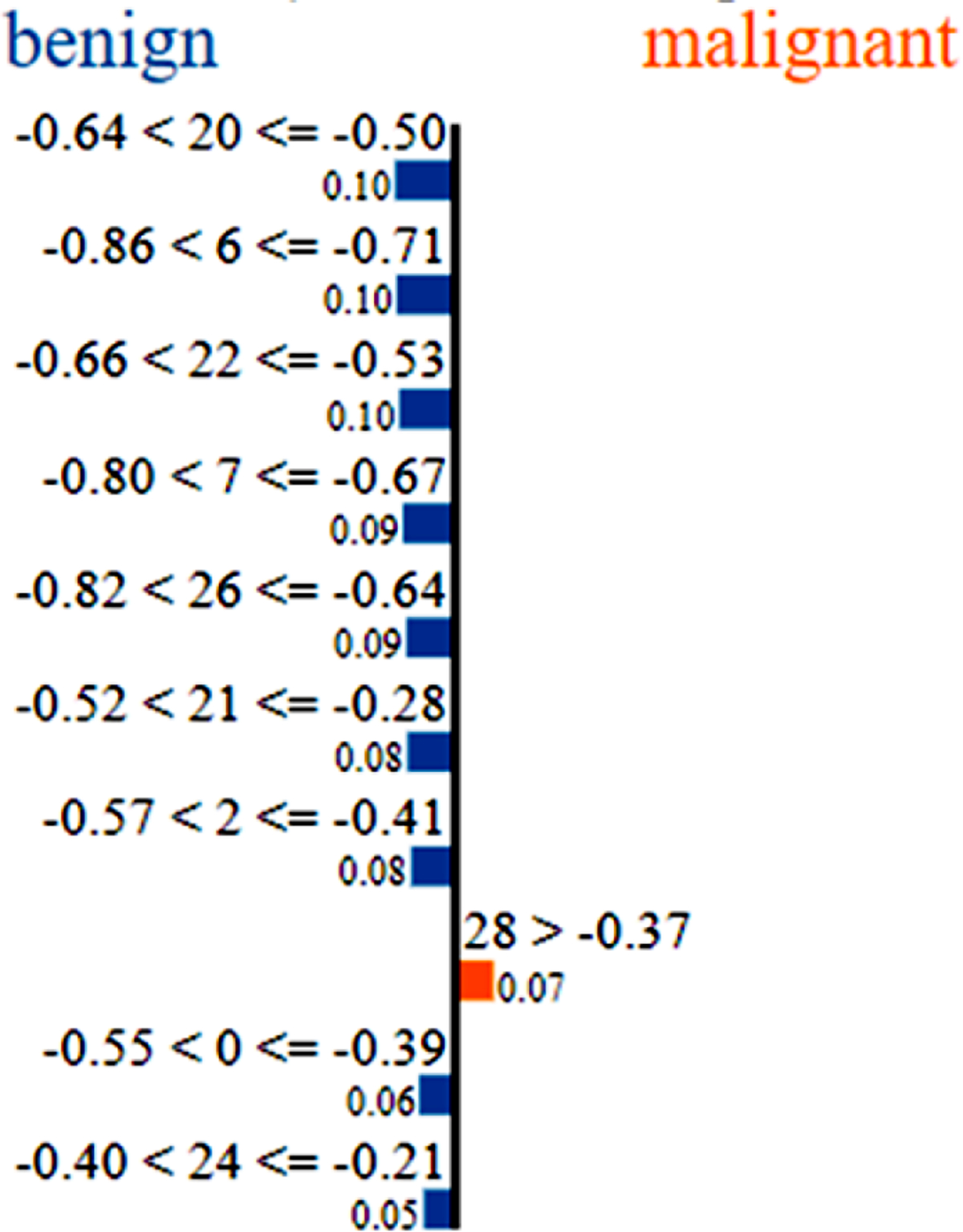
LIME output for a data point which is classified as benign.

LIME is used to explain the predictions made by the DNN on the patients (data points) identified in the test set. The outputs from LIME are shown for two data points from the test set in Figures 3 and 4. The first number above each horizontal bar refers to the index of the input variable. The length of each bar is proportional to the contribution factor of that input variable mentioned next to it. For the data point in Figure 3, inputs 21, 27 and 24 are the three most contributing variables that drive the prediction to malignant with contribution factors of 0.28, 0.22 and 0.18, respectively. There are some variables such as inputs 29, 11, 15, etc. that try to drive the prediction to benign. However, the contributions of these inputs are lower for this particular data point.

For the data point in Figure 4, most of the major input contributions seem to drive the prediction correctly to benign. In this case, inputs 20, 6 and 22 (radius (worst), concavity (mean) and perimeter (worst), respectively) are the most important inputs, each with a contribution factor of 0.1. For these two cases, it is understood that lower values for most of the features indicate benign masses while higher values indicate malignancy. This is consistent with expert understanding of malignant masses^[30]^. The LIME outputs thus help us gain an understanding of the variables and their values that affect the predictions made by the trained DNN.

### 4.2. Results with SHAP

**Figure 5.**
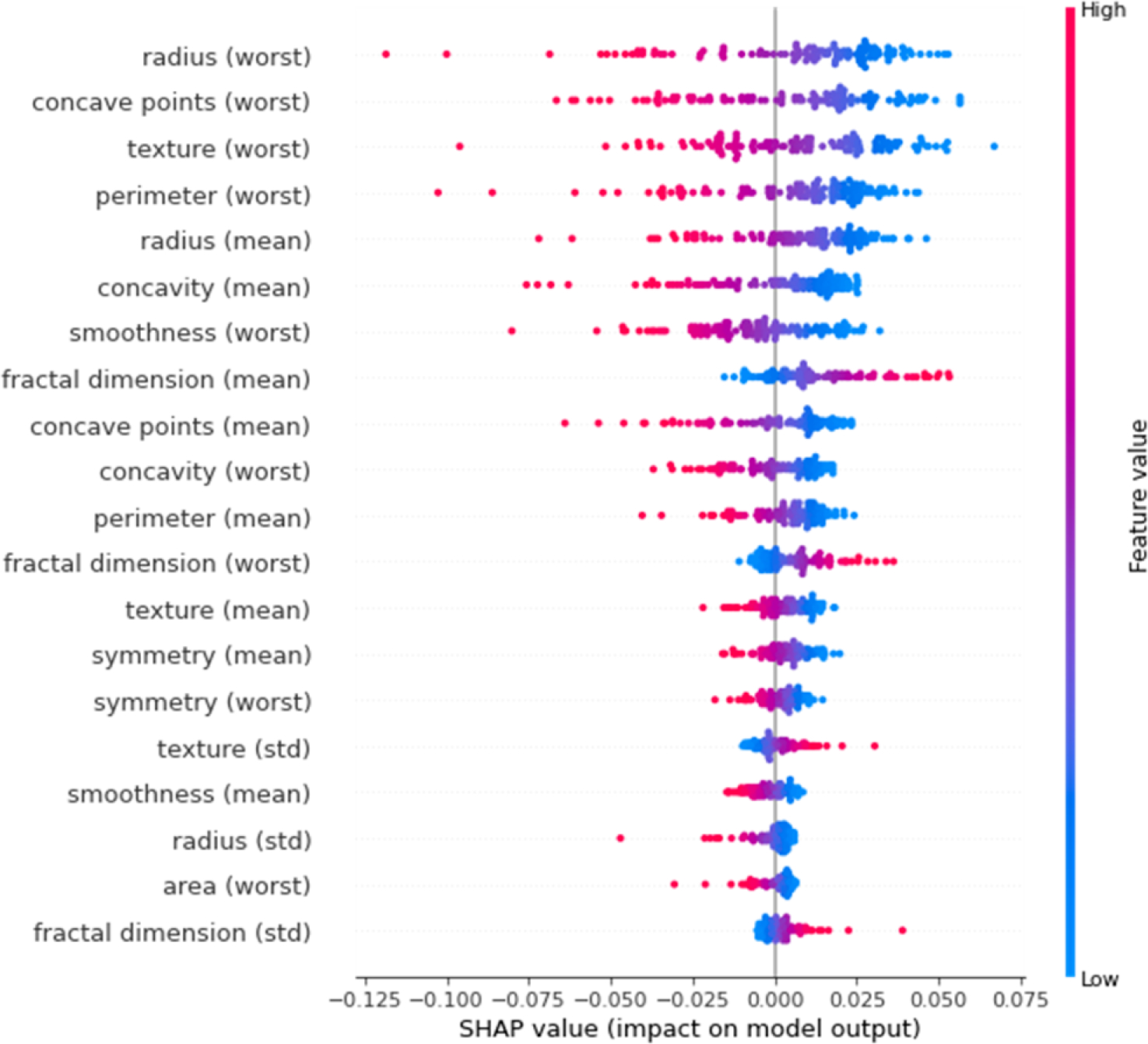
SHAP summary plot on the test data for the benign output class

SHAP was also used to analyze the predictions made by the trained DNN on the data points from the test set. The Shapley values of each input feature can be evaluated for each data point. The mean of the absolute shapley values of each feature across the data can be used to evaluate the importance of the features. Figure 5 shows the summary plot of shapley values across the test data. The shapley values are plotted for the benign output class. Hence, higher shapley values imply higher chances of a benign prediction. The color of the points represents the feature values, with lower values shown by blue and higher values shown by red points. Overlapping points are jittered vertically. The input features are ordered in descending order of importance which is measured using the mean of the absolute shapley values across the data for feature. This can also be noticed from the fact that moving down, the distribution of shapley decreases.

From Figure 5, we can infer that lower values of certain features such as radius (worst), concave points (worst), texture (worst), etc. indicate a benign prediction. On the other hand, higher values for the same features indicate a malignant prediction. This is in line with expert’s understanding of malignancy of breast masses as described in the UCI breast cancer Wisconsin database^[30]^. In fact, the features in this dataset are defined such that higher values indicate malignancy. Additionally, the SHAP summary plot also correctly identifies that the worst values of the different variables are more important for differentiating between benign and malignant masses.

**Figure 6.**
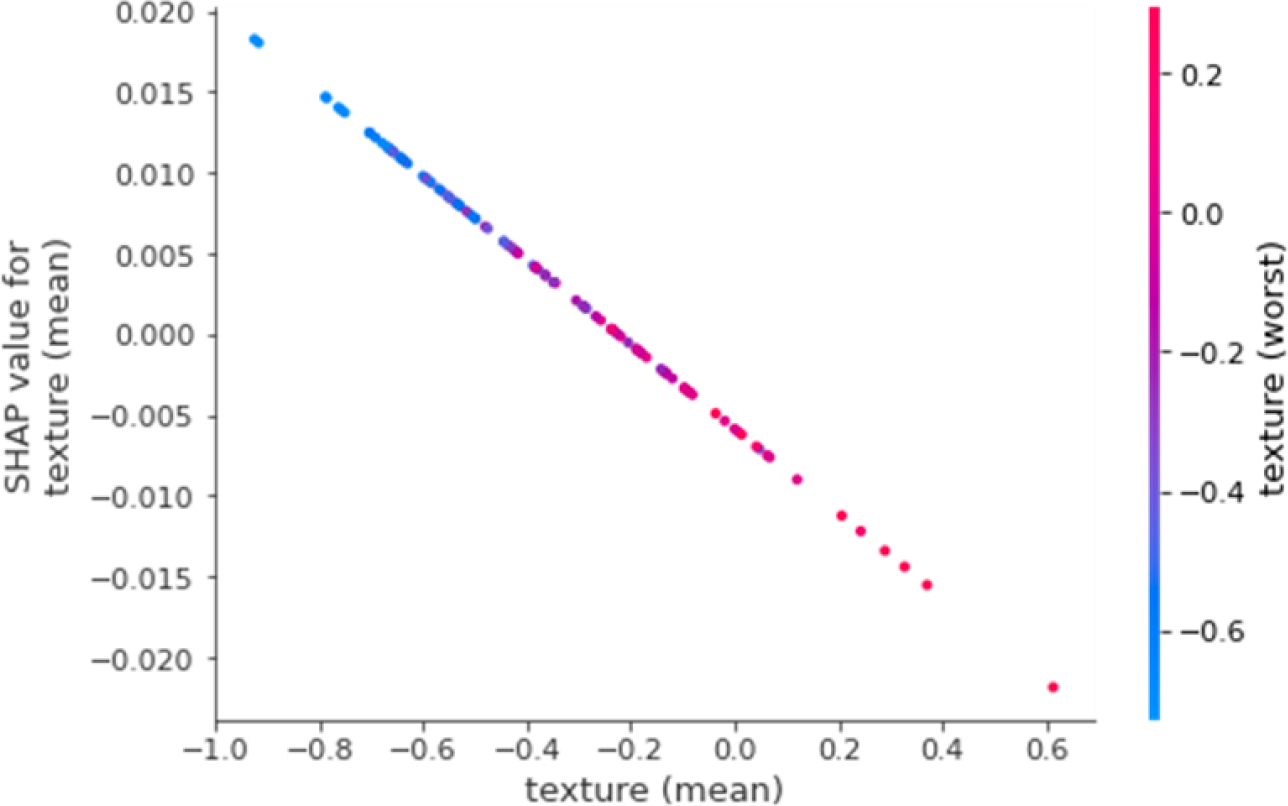
SHAP dependency plot for texture (mean). SHAP: Shapley Additive exPlanations.

SHAP dependency plots can provide additional insights about the dependency between features and their effect on the shapley values. For example, Figure 6 shows the SHAP dependency plot for the input feature texture (mean). This feature has the highest dependency on another input feature texture (worst) and hence is also shown in the plot. It can be noticed that shapley values for texture mean linearly decreases with increasing texture (mean). Additionally, based on the colored points, it can be seen that higher texture (mean) also has higher texture (worst).

**Figure 7.**
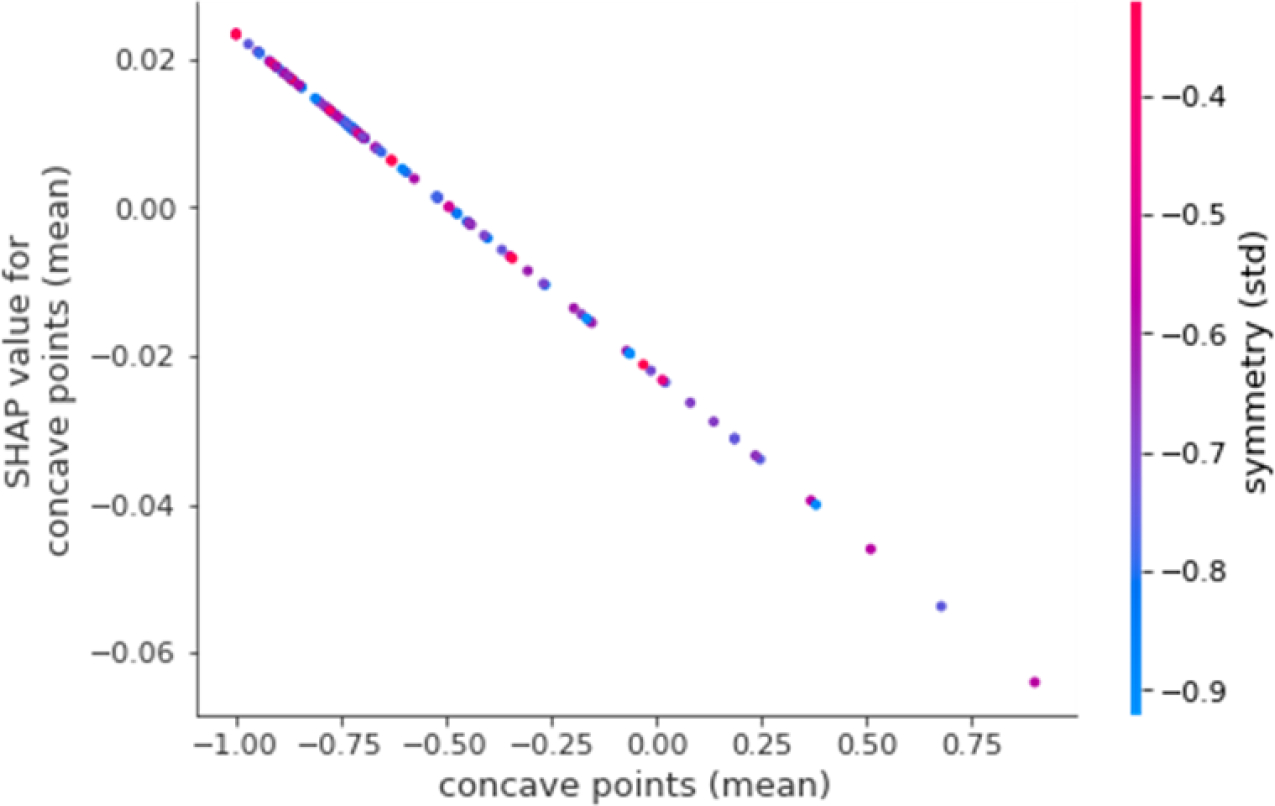
SHAP dependency plot for concave points (mean). SHAP: Shapley Additive exPlanations.

As another example, Figure 7 shows the SHAP dependency plot for the feature concave points (mean). The feature with the highest dependency on this feature is symmetry (std). Again, it can be seen that the shapley values for concave points (mean) linearly decrease with increasing values for the feature concave points (mean). However, symmetry (std) does not necessarily have a linear relationship with the chosen feature, as can be seen from the distribution in the colors of the different points on the plot. We can see points with low and high values of symmetry (std) for lower values of concave points (mean).

Certain commonalities can be found between the SHAP summary plot in Figure 5 and the LIME plots for individual data points from Figures 3 and 4. For example, Figure 3 shows that higher values of texture (worst), smoothness (worst) and concave points (worst) (inputs 21, 24 and 27, respectively) drive that data point to malignant prediction. The same can be noticed from the SHAP summary plot. Similarly, from Figure 4, lower values of radius (worst), concavity (mean) and perimeter (worst) (inputs 20, 6 and 22, respectively) drive that data point towards benign prediction. The same trend can be seen from the SHAP summary plot in Figure 5.

The above analysis suggests that explainability tools such as LIME and SHAP can be invaluable tools in analyzing trained models and understanding their predictions. These tools can help us obtain trends in the predictions from the trained models to explain the decisions made by the model. LIME and SHAP could be used for multi-class classification (with more than two classes)^[31]^, regression^[32]^ and other types of applications such as image processing using CNNs^[33]^, etc. Since both tools have to run the trained model several times to produce explanations, it may not be useful for real-time explanations. The computational complexity of methods would depend on the computational time needed to make inferences. For example, larger neural networks could be more complicated to use as inputs to LIME and SHAP. However, they can still be a valuable tool for obtaining explanations for applications that do not require real-time explanations or those that only require explanations during certain instances.

## 5. CONCLUSIONS AND FUTURE WORK

In this paper, we presented the use of two explainability tools, namely LIME and SHAP, to explain the decisions made by a trained DNN model. We used the popular Breast Cancer Wisconsin dataset from the UCI repository as the use case for our work. We presented the trends obtained using LIME and SHAP on the predictions made by the trained models. The LIME outputs were shown for individual data points from the test data. On the other hand, SHAP was used to present a summary plot that showed a holistic view of the effect of the different features on the model predictions across the entire test dataset. Additionally, the paper also presented common trends between the analysis results from both LIME and SHAP.

For future work, we plan to use these tools for other datasets, especially those with more than two output classes. It will be interesting to see how the results from LIME and SHAP analysis can help gain insights into datasets with a larger number of classes. The results from this paper are very encouraging to the research efforts on advancing explainability to deep learning based machine learning models. We also plan to make use of the abstract features derived within the DNN as possible input to LIME and SHAP. This may also help to understand the relevance of abstract features and may be useful for other aspects of machine learning, such as transfer learning.

### 5.1. Note

The Python code is available at this GitHub repository: https://github.com/sathyaa3p/xaiBreastCancer

## Data Availability

Not applicable.
